# High photosynthetic plasticity may reinforce invasiveness of upside-down zooxanthellate jellyfish in Mediterranean coastal waters

**DOI:** 10.1371/journal.pone.0248814

**Published:** 2021-03-19

**Authors:** Marta Mammone, Christine Ferrier-Pagés, Silvia Lavorano, Lucia Rizzo, Stefano Piraino, Sergio Rossi

**Affiliations:** 1 Dipartimento di Scienze e Tecnologie Biologiche ed Ambientali, University of Salento, Lecce, Italy; 2 Centre Scientifique de Monaco, Ecophysiology Team, Monaco, France; 3 Acquario di Genova, Genova, Italy; 4 Department of Integrative Marine Ecology, Stazione Zoologica Anton Dohrn, Napoli, Italy; 5 CoNISMa, Consorzio Nazionale Interuniversitario per le Scienze del Mare, Rome, Italy; 6 Labomar, Universidade Federal do Ceará, Fortaleza, Brazil; Mount Allison University, CANADA

## Abstract

Ecological profiling of non-native species is essential to predict their dispersal and invasiveness potential across different areas of the world. *Cassiopea* is a monophyletic taxonomic group of scyphozoan mixotrophic jellyfish including *C*. *andromeda*, a recent colonizer of sheltered, shallow-water habitats of the Mediterranean Sea, such as harbors and other light-limited, eutrophic coastal habitats. To assess the ecophysiological plasticity of *Cassiopea* jellyfish and their potential to spread across the *Mare Nostrum* by secondary introductions, we investigated rapid photosynthetic responses of jellyfish to irradiance transitions—from reduced to increased irradiance conditions (as paradigm of transition from harbors to coastal, meso/oligotrophic habitats). Laboratory incubation experiments were carried out to compare oxygen fluxes and photobiological variables in *Cassiopea* sp. immature specimens pre-acclimated to low irradiance (PAR = 200 μmol photons m^**−**2^ s^**−**1^) and specimens rapidly exposed to higher irradiance levels (PAR = 500 μmol photons m^**−**2^ s^**−**1^). Comparable photosynthetic potential and high photosynthetic rates were measured at both irradiance values, as also shown by the rapid light curves. No significant differences were observed in terms of symbiont abundance between control and treated specimens. However, jellyfish kept at the low irradiance showed a higher content in chlorophyll *a* and *c* (0.76±0.51SD vs 0.46±0.13SD mg g^-1^ AFDW) and a higher Ci (amount of chlorophyll per cell) compared to jellyfish exposed to higher irradiance levels. The ratio between gross photosynthesis and respiration (P:R) was >1, indicating a significant input from the autotrophic metabolism. *Cassiopea* sp. specimens showed high photosynthetic performances, at both low and high irradiance, demonstrating high potential to adapt to sudden changes in light exposure. Such photosynthetic plasticity, combined with *Cassiopea* eurythermal tolerance and mixotrophic behavior, jointly suggest the upside-down jellyfish as a potentially successful invader in the scenario of a warming Mediterranean Sea.

## Introduction

Jellyfish are among the most versatile marine invertebrates. They can form large aggregations, thanks to a variety of reproductive and trophic strategies that allows a rapid increase in their abundance [1–3]. During population outbreaks, jellyfish can be strong controllers of food webs and energy flux in marine ecosystems. They can exert a top-down control through direct predation on multi-level consumers, including sensitive fish life stages (e.g., eggs, larvae and juveniles) [4] and/or a bottom-up control by diet competition and food limitation to upper trophic levels [5–8]. Nowadays, direct anthropogenic stress (e.g., overfishing, coastal pollution, coastal mismanagement) together with ocean warming may contribute to shifting some coastal ecosystems towards increasing jellyfish populations, especially medusozoans, ctenophores, and thaliaceans [9–11].

Jellyfish blooms can be even more damaging when alien species are involved [5] both affecting marine biodiversity through predation and competition [7] and causing economic damages on several human activities in the coastal zone (e.g., tourism, coastal industry, fishery and aquaculture) [12, 13]. Jellyfish such as *Cassiopea* spp., *Phyllorhiza punctata*, *Rhopilema nomadica* are some of the successful non-native species colonizing the Mediterranean Sea (reviewed by Bayha and Graham [14]). The invasiveness of a species inherently depends on its life history, environmental tolerance and trophic strategy [14]. Trophic plasticity is renowned in several marine invertebrates–including sponges, corals, and jellyfish–due to their mutualistic relationships with single-celled, dinoflagellate algae belonging to the family Symbiodiniaceae [15, 16]. Out of over two hundred and twenty taxa of scyphozoan jellyfish are recognized so far [17], nearly 20–25% of them (mostly belonging to the rhizostomid Kolphophorae sub-order) having symbiotic associations (obligatory or facultative mutualism) with *Symbiodium* spp. and other Symbiodiniaceae dinoflagellates. These include several rhizostome species, such as the Mediterranean *Cotylorhiza tuberculata* [18], 10 different species of upside-down jellyfish (*Cassiopea* spp.) [19, 20], the spotted jellyfish *Phyllorhiza punctata* [21], and the golden jellyfish *Mastigias papua*, where up to 10% of its protein biomass is produced by their endosymbiotic dinoflagellates [22]. This mutualistic association grants jellyfish a mixotrophic strategy (i.e., based on concurrent heterotrophic and autotrophic energy inputs) [23]. Mutualism is realized by jellyfish providing a sheltered habitat—rich in nitrogen and phosphorus catabolites—to microalgae that, in turn, release organic carbon products (e.g., sugars) to their host [24, 25]. Mixotrophy may be considered as the dominant feeding strategy for symbiotic jellyfish, however the relative importance of autotrophic versus heterotrophic nutrition is variable not only among species, but also within each species’ life cycle and depending on seasonality [23]. In most cases, photosynthesis largely covers the respiratory needs, and assimilation of additional inorganic nutrients is required from the surrounding water column [26–28]. The benthic behavior and bell pulsation of the upside-down jellyfish were suggested to facilitate mobilization of nutrients from the sediment pore water, naturally enriched through the degradation of settling organic matter [29]. Although mixotrophy is a widespread phenomenon [30], the extent of the relative contribution of autotrophy and heterotrophy to the energetic budget of the organisms is challenging to quantify. In particular, available information about mixotrophy in scyphozoans is scant [20, 25, 31, 32]. In this framework, knowledge of the ecophysiology and trophic strategies of jellyfish may contribute to understanding the mechanisms driving the structure and organization of marine communities in a warming ocean scenario [33].

Commonly known as the upside-down benthic jellyfish, *Cassiopea* includes a group of 10 species native of tropical and sub-tropical, shallow-water environments, as the majority of jellyfish taxa associated with Symbiodiniaceae dinoflagellates [34, 35]. However, global sea warming boosted the natural spread as well as the human-mediated translocation of *Cassiopea* spp. to new areas. *Cassiopea andromeda* is the earliest known lessepsian jellyfish in the Mediterranean Sea and reached the basin in the early 20th century [36]. Indeed, the progressive enlargement of the Suez Canal caused the progressive dilution of the natural hyperhaline barriers of the Bitter Lakes [37] bolstering the northern migration of Red Sea species, so that today the Suez Canal can be considered the most potent corridor of marine bioinvasion in the world [38]. Therefore, the indopacific *C*. *andromeda* moved into the Mediterranean Levant Sea by an anti-clockwise migration from Israel and Lebanon [39, 40], to Cyprus, Turkey and Greece [41–43] where its populations can form short-term outbreaks up to 20 individuals m^-2^. Later, *C*. *andromeda* was observed with an episodic but increasingly common occurrence further west in the Mediterranean: in 2009, *C*. *andromeda* was spotted in Maltese islands [44] and shortly after in Sicily [45, 46]. The northern limit of dispersal of many Lessepsian immigrants, including the upside jellyfish, across natural environments is controlled by the winter sea surface temperature, and currently coincident with the winter position of the 15°C isotherm [47–49].

Due to a predominantly benthic lifestyle, *Cassiopea* jellyfish live in shallow habitats with reduced water movements, such as sandy mudflats, mangroves, estuaries, harbors and artificial coastal sounds, laying down on the sea floor on the exumbrellar side, with an upside orientation of oral arms extended into the water column [50, 51]. These areas are characterized by nutrient-enriched and shaded environments, due to significant anthropogenic nutrient loads and high turbidity [52, 53]. The photosynthetic active radiation (PAR) reaching the sea floor may be locally limited (< 100 μmol photons m^**−**2^ s^**−**1^) in transitional habitats with high anthropogenic inputs, such as in the harbor of Ischia (gulf of Naples) or in the Venice lagoon [54, 55]. Conversely, in nearby but less shaded areas irradiance can be much higher (260–1760 μmol photons m^**−**2^ s^**−**1^) [55].

Photobiology of zooxanthellate jellyfish has been studied in only a few species from tropical areas, including *Mastigias* sp., *Linuche unguiculata* and a tropical conspecific of *Cassiopea* [20, 29, 56]. Welsh et al. [56] investigated the rates of photosynthesis of *Cassiopea* spp. collected in a tropical mangrove of Queensland (Australia), where light ranges between 200 and 2000 μmol photons m^-2^ s^-1^ depending on the time of the day. Saturation of photosynthesis was found at an irradiance of 400 μmol photons m^-2^ s^-1^ while the photosynthetic compensation was achieved at an irradiance of 50 μmol photons m^-2^ s^-1^. In addition, higher photosynthesis rates were observed in summer than winter, and proportional to the light level and thermal regimes [20]. Studies on *Mastigias papua* (Palau) and *L*. *unguiculata* (Bahamas) showed that their photosynthetic systems respectively reached saturation at 307–416 or 200–300 μmol photons m^-2^ s^-1^, and a compensation irradiance of 53–63 or 20 μmol photons m^-2^ s^-1^, [31, 32]. However, *C*. *andromeda* seems to have a high photosynthetic efficiency also under high light intensities, as experienced in its native habitat (the Red Sea) where irradiance may reach up to 1000 μmol photons m^-2^ s^-1^ at noon [29]. In this framework, assessing the photosynthetic performance of *Cassiopea* in the Mediterranean Sea—where light might be a limiting factor compared to native areas–will be key to the ecological profiling of the upside-down jellyfish, required to establish its dispersal and invasiveness potential. Eutrophic and artificial environments, such as harbors and marinas and coastal lagoons, are known to be primary terminals of immigrant alien species from where bioinvasions may start, by natural spillover into the surrounding, mesotrophic open waters or by secondary, human-mediated spread in other areas, facilitating settlement of zooxanthellate jellyfish populations, as demonstrated by the records of *C*. *andromeda* in the central Mediterranean Sea (reviewed by Deidun et al. [57]). To counterbalance the parallel reduction of light penetration in eutrophic waters, zooxanthellate jellyfish exhibit evolutionary adaptive mechanisms, such as *M*. *papua* swimming in shallower waters with a circadian rhythm [58] or settling at shallow depths, as for the benthic *Cassiopea* jellyfish.

In the present work we investigated the short time reaction in the mixotrophic strategy of *Cassiopea* switching from a simulated eutrophic (low light, harbor-like) to meso/oligotrophic (open water-like) conditions in order to explore its rapid acclimation potential. Photosynthesis measurements of *Cassiopea* sp. exposed to different light intensities were carried out together with Pulse Amplitude Modulated (PAM) measurements. These data were integrated with the measurements of the endosymbiotic algal density, total chlorophyll and protein concentrations to frame the key elements of *Cassiopea* autotrophic strategy with the ultimate aim of exploring the invasiveness potential of this zooxanthellate jellyfish in new environments.

## Materials and methods

### Acclimation

Bred at the plankton laboratory of the Acquario di Genova (COSTA Edutainment), twenty *Cassiopea* sp. jellyfish were acclimated to a baseline condition at the Centre Scientifique de Monaco research facility one month before the start of the experiment. Abiotic factors were set to simulate a turbid and nutrient-enriched environment: particularly, irradiance was set at 200 μmol photons m^**−**2^ s^**−**1^ and jellyfish were fed *ad libitum* once a day with *Artemia salina* nauplii and ground fish meat. The starting PAR intensity (200 μmol photons m^**−**2^ s^**−**1^) was chosen based on irradiance data of eutrophic areas such as harbors and lagoons [54, 55].

### Experimental design

Five jellyfish were reared into each of four different tanks, which were continuously supplied with natural seawater pumped through a pipeline directly from the sea, at a flow rate of 10 L h^**−**1^, also equipped with a filtration system with a sand filter and an external biological filter with plastic bio balls. This ensured constant pH, salinity, dissolved oxygen concentrations, and removal of excess nitrogen and nitrites. Seawater was set up to 24°C ± 0.2°C (summer Mediterranean temperature) using computer-controlled electronic heaters.

Two aquaria were maintained at the starting irradiance of 200 μmol photons m^**−**2^ s^**−**1^ (on a 12 h light: 12 h dark photoperiod), while the two others were set at 500 μmol photons m^−2^ s^−1^, using 400 WHQI metal halide lamps. The tested PARs were chosen based on average irradiance values found in eutrophic vs meso/oligotrophic areas [54, 59] and on the evidence that the irradiance may considerably increases from inside to outside harbor waters (up to five times [55]). From here on, irradiance equals to 200 μmol photons m^**−**2^ s^**−**1^ will be referred as RLP (reduced light penetration) whereas ELP (enhanced light penetration) will stand for irradiance equaling 500 μmol photons m^**−**2^ s^**−**1^. Jellyfish were maintained at either RLP or ELP irradiance conditions one week before the following experimental measurements.

### Photosynthesis measurements

Oxygen fluxes were quantified using temperature-regulated Plexiglas chambers filled with 0.22 μm-filtered seawater and equipped with Unisense optodes connected to a computer with OXY-4 software (channel fibre-optic oxygen meter, PreSens, Germany). Optodes were calibrated against N_2_-saturated and air-saturated seawater for 0% and 100% oxygen saturation respectively. Oxygen fluxes for the estimation of net photosynthesis (Pn) were monitored over 10 to 15 min (in 1 min intervals) at 200, 400, 600, 800, 1000, 1200 and 1400 μmol photons m^–2^ s^–1^. Respiration rates (R) were quantified through a dark incubation period of 20 min before (dark respiration, R_d_) and after the last light stimulation (post-illuminatory respiration, R_L_). At the end of the incubation, the volume of water inside the chamber was recorded upon jellyfish removal. Pn and R rates were calculated by regressing oxygen data against time and normalized to ash free dry weight (AFDW), as described below. Gross photosynthesis (Pg) was calculated as the sum of |Pn| and |R| and was plotted versus irradiance (P/E curves, formerly P/I [60]). Photosynthesis to respiration ratio (P/R) was calculated by considering 12 h light at saturating irradiance and 24 h of respiration. The highest photosynthetic rate was considered as P_max_, while the photosynthetic efficiency (α) was calculated from the initial slope in the light-limiting region of the P-I curve [61]. The theoretical saturation irradiance (E_k_) was calculated as the intercept between the initial slope and the horizontal asymptote (P_max_/α) [62]. The compensation irradiance (E_c_), where the oxygen flux is zero (Pn = 0) was as well obtained from the P-I curves.

A pulse amplitude modulated fluorometer (DUAL-PAM/F, Waltz, ®) was used to further investigate *Cassiopea* photosynthetic performances according to Ralph and Gademann [61] after the jellyfish were maintained 20 min in the dark. The relative electron transport rate (rETR), representing an approximation of the rate of electrons pumped through the photosynthetic chain [63], was assessed using rapid light curves (RLCs). The effective quantum yield (Y) and the rETR were recorded from the upward-oriented sub-umbrella (i.e., the concave oral side) and oral arms of the jellyfish, with 10 seconds steps of irradiance from 0 to 1956 μmol photons m^–2^ s^–1^. Non-photochemical quenching (NPQ), which represents a measure of the heat-dissipation of the excess energy absorbed by the photosystem II was as well recorded during RLCs. After incubations, Falcon tubes containing the experimental jellyfish were immersed in liquid nitrogen and then stored at -80°. Samples were freeze-dried and the powder was used for different measurements. All applicable international, national, and/or institutional guidelines for the care and use of animals were followed in accordance with the ethical standards of the European Union (Directive 609/86).

### DNA extraction

To characterize the symbiont genotype associated with the jellyfish, we investigated the chloroplast (cp) subunit rDNA sequence (Cp23S-rDNA) [64]. 18 to 20 mg of freeze-dried powder was used for DNA extraction using the DNeasy Plant Mini Kit (Qiagen) according to manufacturer’s instructions. Primers 23S1M13 (5’-CACGACGTTGTAAAACGACGGCTGTAACTATAACGGTCC-3’) and 23S2M13 (5’-GGATAACAATTTCACACAGGCCATCGTATTGAACCCAGC-3’), were used [64]. After performing PCR and agarose gel electrophoresis, bands of DNA were cut out from the gel and DNA was purified using MinElute Gel Extraction Kit (Qiagen).

### Symbiont and chlorophyll quantification

A known amount (ranging 300–500 mg) of the total freeze-dried powder was resuspended in 10mL of distilled water and homogenized for 20 min with a potter tissue grinder. The total volume of the slurry was recorded. A subsample of 1 mL was stored at +4°C for the later determination (within few hours) of the symbiont concentration, using a polarizing light microscope Leica DM750P and a counting chamber (Neubauer-improved). The remaining slurry was centrifuged at 3000*g* for 10 min, at 4°C, to pellet the symbionts and extract the chlorophyll pigments. The supernatant was thus removed and the pellet was re-suspended in 100% acetone (10mL), mixed with a vortex stirrer and let for 24 h covered with aluminum foil at +4°C. Samples were then centrifuged for 15 minutes, at 10,000 *g* and 4°C and then analyzed using a SAFAS UVmc2 double-beam UV-visible spectrophotometer. The absorbance was read at 630, 663, 750nm. Chlorophyll *a* and *c*_*2*_ concentration were calculated according to the spectrometric equations reported in Jeffrey and Humphrey [65]. Total chlorophyll was computed as the sum of chlorophyll *a* and *c*_*2*_. The chlorophyll content per symbiont cell (Ci) was computed as the ratio between the chlorophyll and the number of symbionts.

### Protein content

A known amount of powder (ranging 10–50 mg) was resuspended in 500 μL distilled water as described above. An equivalent volume of sodium hydroxide (NaOH) was added before samples were incubated in the oven at 60°C for five hours. Proteins were measured by the INTERCHIM protein quantitation kit (following [66], with bovin serum albumin as a standard and NaOH as blank), using a SAFAS XeniusXM spectrofluorometer.

### Data normalization

A fraction of the dry powder of each sample was inserted into an aluminum cup and the weight was determined using a precision balance. Samples were heated in an oven for 4 h at 430°C. Ashes were re-weighted in order to calculate the ash free dry weight (AFDW = DW-AW) and normalize the data.

### Statistical analysis

All data were tested for assumption of homogeneity by the Cochran test and transformed when needed (log (x+1)). All the data were tested using one-way ANOVA (analysis of variance). Two-ways ANOVA was used to test the effects of RLCs. Statistical analyses were computed using STATISTICA® software (StatSoft, Tulsa, USA). All the data are expressed as mean and standard deviation (SD). Graphs and correlation coefficients (Pearson’s) were obtained by using Kaleidagraph (Synergy Software, Reading, PA).

## Results

### Symbiont classification/taxonomy, density and chlorophyll content in *Cassiopea*

**Fig 1:**
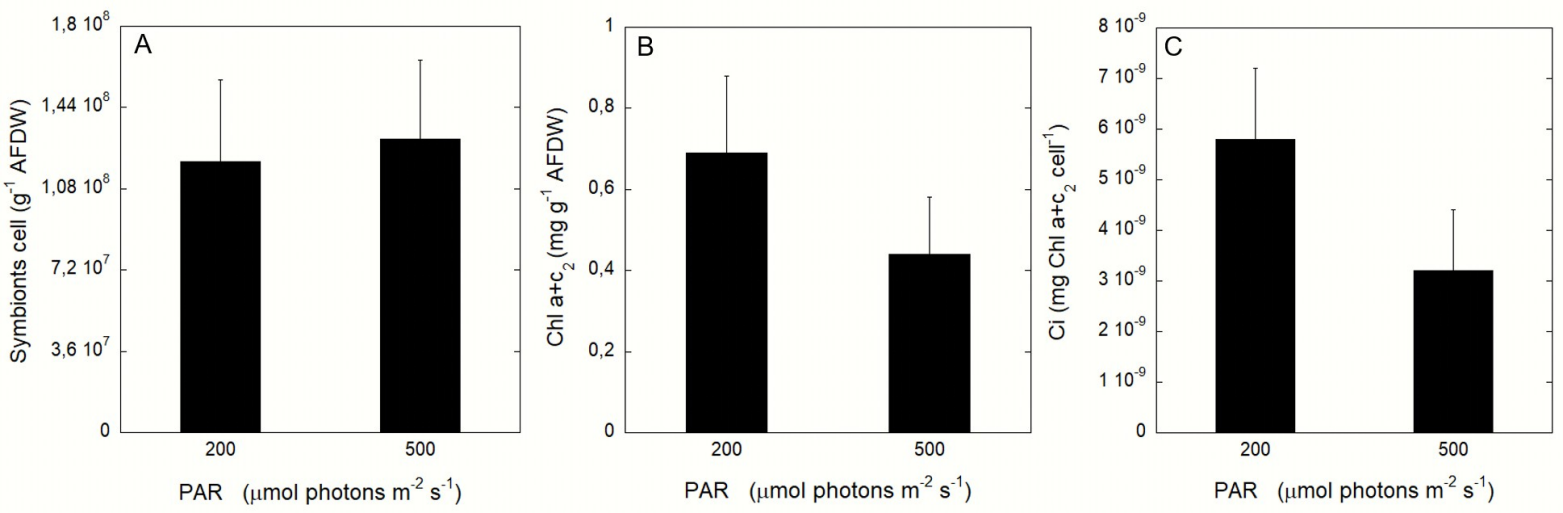
*Cassiopea* symbionts density and chlorophyll content. (A) Symbiont density (B) Total chlorophyll (a+c_2_) and (C) Chlorophyll content per symbiont, under the two different light conditions (RLP and ELP). Data are normalized to AFDW. Each point represents mean *±* SD, n = 20.

All jellyfish specimen (n = 20) hosted the Symbiodiniaceae *Cladocopium* sp. (Clade C). No significant difference in symbiotic cells density was observed between the RLP or ELP treatments (F = 0.7412, p>0.05). The mean symbiont density was 1.3_*_10^8^ ±0.37_*_10^8^ (SD) and 1.2_*_10^8^ ± 0.36*10^8^ (SD) cell g^-1^ AFDW for RLP and ELP, respectively (Fig 1A). Conversely, total chlorophyll content normalized to ash-free dry weight (AFDW) varied significantly (F = 8.9532, p< 0.01) between the two irradiance conditions. The ELP condition was associated with a lower Chl content = 0.46±0.13 (SD) mg g^-1^ AFDW, compared to the RLP condition (Chl content = 0.76±0.51 (SD) mg g^-1^ AFDW) (Fig 1B). Higher content of chlorophyll *a* than chlorophyll *c* (RLP: F = 13, p<0.001; ELP: F = 19, p<0.01) occurred at both irradiances (S1A and S1B Fig). Chlorophyll content per symbiont cell (Ci) was higher (F = 17, p<0.01) for the jellyfish from the RLP (Fig 1C). The same trend in symbionts density and chlorophyll content was observed using DW as normalization (S1C–S1E Fig).

### Proteins and organic matter in *Cassiopea*

**Fig 2:**
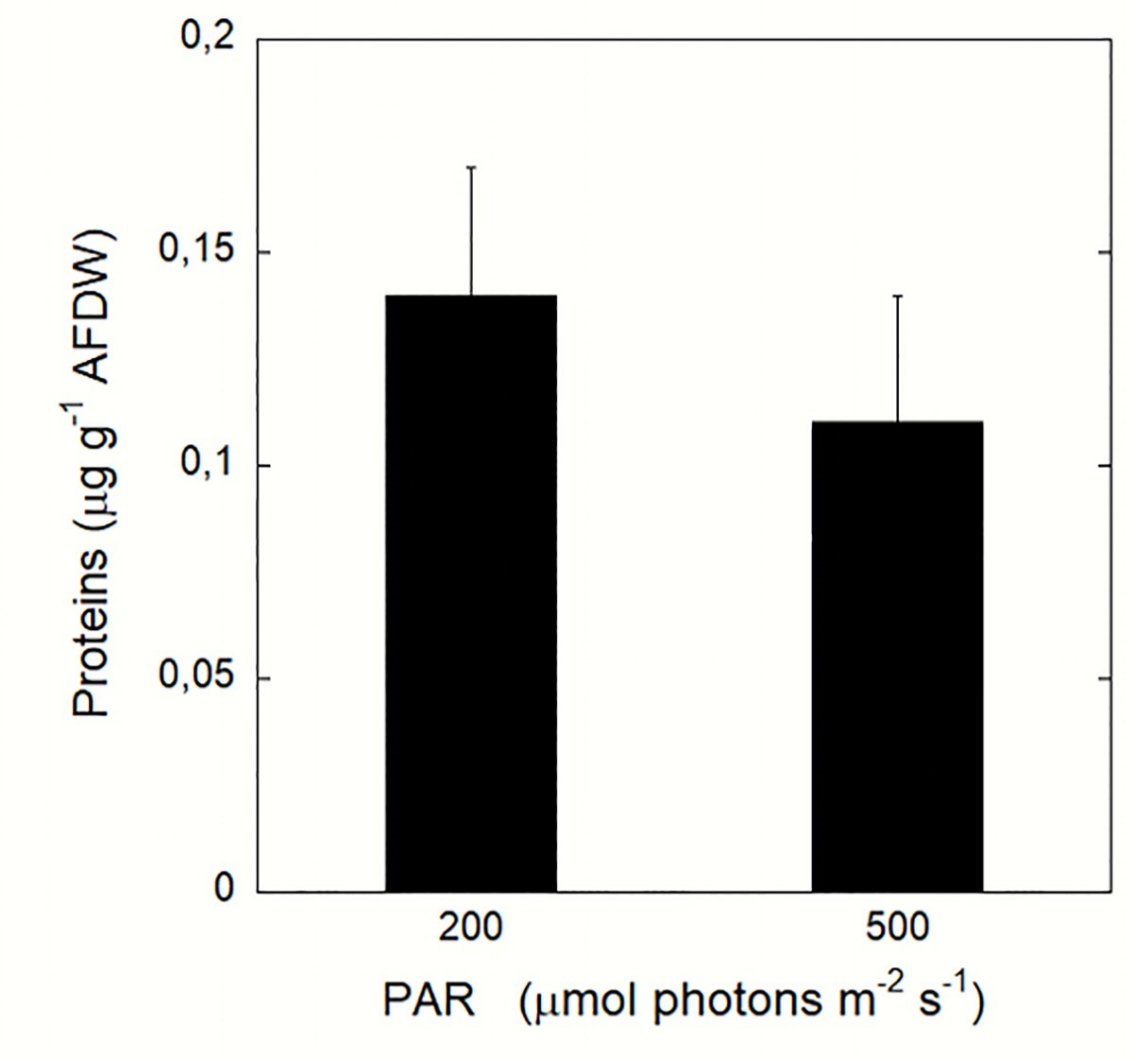
Protein content of *Cassiopea*. Protein content is expressed as μg per g^-1^ of AFDW for each light condition (RLP and ELP). Each point represents mean*±* SD, n = 20.

A significant difference (F = 16.169, p<0.001) was observed in the organic matter (OM) content which was 27% ±3 (SD) and 33% ± 4 (SD) for the jellyfish exposed to the RLP and ELP irradiance conditions, respectively. The protein content was in the same range in all samples: 0.11 ± 0.03 (SD) μg g^-1^ AFDW in ELP-treated jellyfish and 0.14 ± 0.03 (SD) μg g^-1^ AFDW in RLP-treated jellyfish, but with significant differences between the RLP and ELP conditions (F = 8.6904, p<0.01) (Fig 2).

### Photobiological performance of *Cladocopium-Cassiopea* association

#### Photosynthesis to irradiance curves

**Fig 3:**
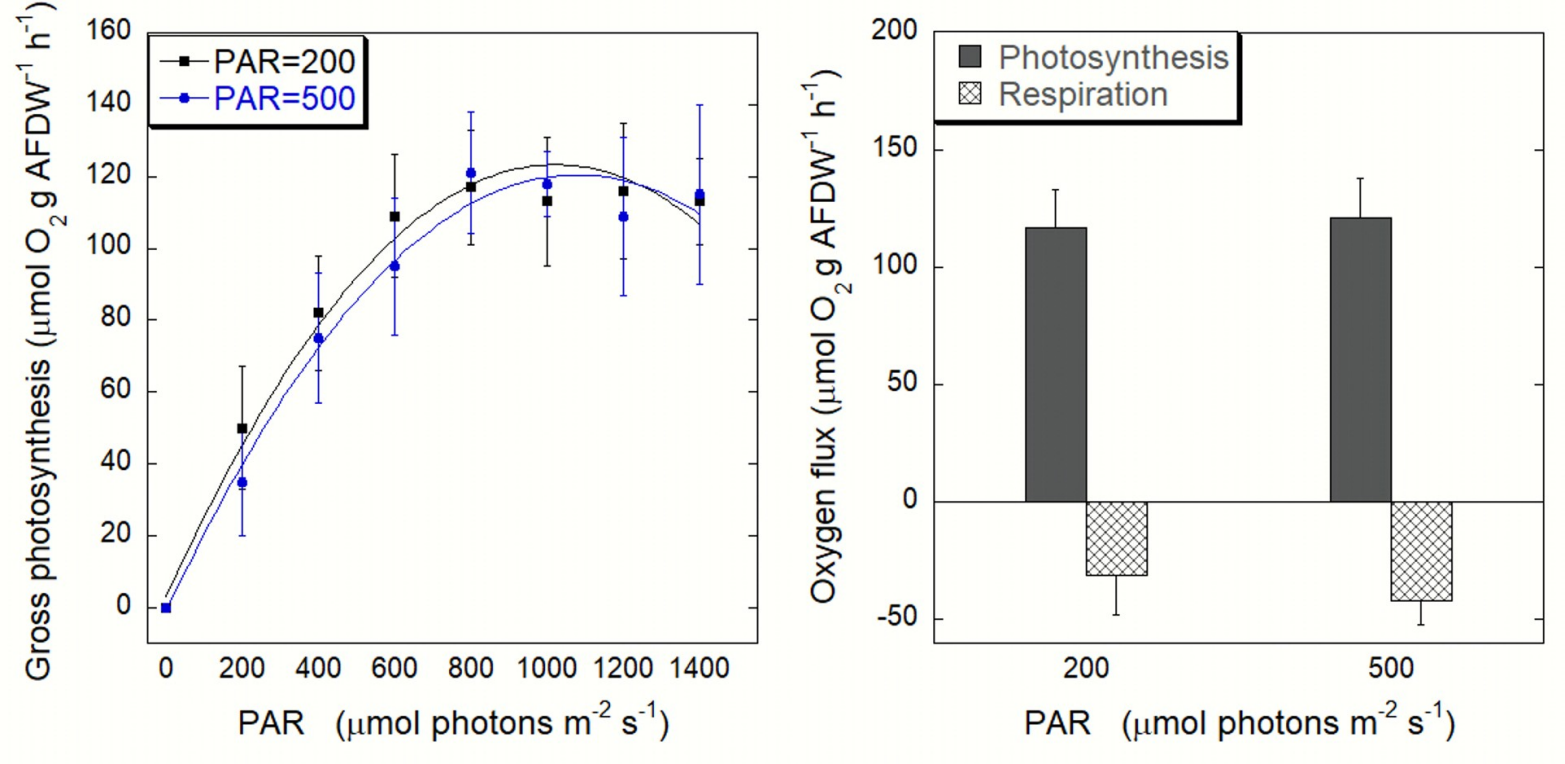
*Cladocopium*-*Cassiopea* photobiological performance. (A) Gross photosynthesis versus irradiance and (B) gross photosynthesis at saturating irradiance (800 μmol photons m^-1^ s^-1^) and dark respiration of the two RLP and ELP conditions. Data are normalized to AFDW. Each point represents means *±* SD, n = 20.

The oxygen flux increased with irradiance (Fig 3A, PAR 200 R^2^ = 0.987; PAR 500 R^2^ = 0.988). When comparing the photosynthetic performances of jellyfish exposed to the two irradiance conditions, no significant differences were observed relative to their maximal rates of photosynthesis (P_max_), efficiency (α) and theoretical saturation irradiance (E_k_) (Table 1). A slight decrease (photoinhibition) was observed in jellyfish maintained at both RLP and ELP experimental conditions, when approaching 1200 μmol photons m^-2^ s^-1^. Compensation irradiance (E_c_) was 196 (PAR = 200) and 238 (PAR = 500) μmol photons m^-1^ s^-1^. Net photosynthesis followed a similar trend (S2 Fig). No significant differences (F = 3.0911, p>0.05) in dark respiration rates were observed between the RLP (PAR = 200) and ELP (PAR = 500) conditions (Fig 3B).

**Table 1:** Summary of the one-way ANOVA testing different photosynthetic parameters (O_2_ measurements) on *Cladocopium*-*Cassiopea* association under different light conditions.

Parameter	RLP	ELP	p-value
Pmax	125±12	120±14	>0.05
α	0.18±0.02	0.16±0.03	>0.05
Ek,	689±50	748±70	>0.05

ELP (= 500 μmol photons m^–2^ s^–1^); RLP (= 200 μmol photons m^–2^ s^–1^). P_max_ = maximal photosynthetic rate, E_k_ = saturation irradiance, α = photosynthetic efficiency. Data expressed as mean value ± SD. P_max_ and E_k_ expressed as μmol photons m^–2^ s^–1^.

#### Rapid light curves

**Fig 4:**
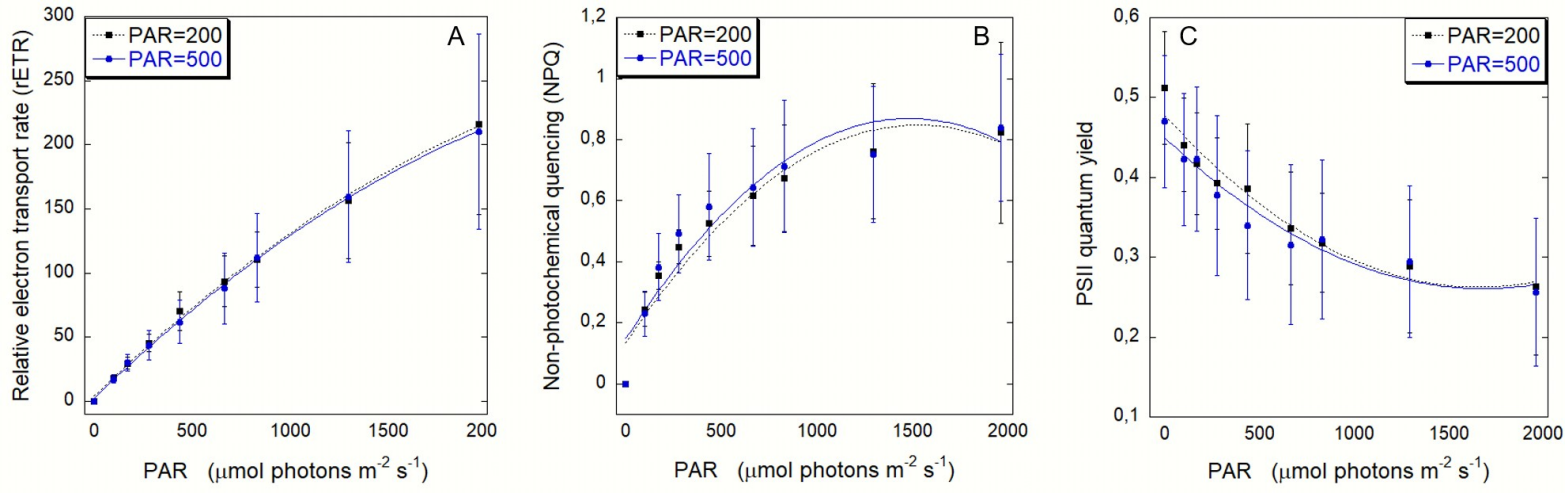
Rapid light curves (RLCs). (A) Relative electron transport rate (rETR) (B) Non-photochemical quenching (NPQ) (C) Yield of PSII (Y) of *Cladocopium*-*Cassiopea* association at the different light conditions (RLP and ELP). Data expressed as mean value *±* SD, n = 20.

No significant difference was found concerning the rETR (F = 1.37, p>0.05; Fig 4A), NPQ (F = 0.678, p>0.05; Fig 4B) and Y (F = 1.447, p>0.05; Fig 4C) at the two experimental conditions. Jellyfish exposed to both conditions showed no saturation also under high light intensities. rETR showed a linear increase at irradiance conditions up to 800 μmol photons m^-1^ s^-1^ (PAR 200 R^2^ = 0.998; PAR 500 R^2^ = 0.999), while at higher light intensities the rETR increased at reduced rate, almost asymptotically (Fig 4A). The NPQ values (Fig 4B) showed a quick increase at RLP but slowed down at ELP irradiance conditions (PAR 200 R^2^ = 0.932; PAR 500 R^2^ = 0.902). The photosynthetic Y of photosystem 2 (Fig 4C) showed a decrease with increasing PAR (PAR 200 R^2^ = 0.952; PAR 500 R^2^ = 0939). When considering the sub-umbrella for PAM measurements, no differences in the NPQ (F = 5, p<0.05) were observed, while ETR (F = 0.19, p>0.05) and Y (F = 6, p<0.01) showed significant differences between the two irradiance conditions (S3 Fig).

## Discussion

*Cassiopea* is well adapted to a highly seasonal environment like the Mediterranean Sea. In the present study, for the first time, the potential autotrophic strategy of *Cassiopea* sp. adapted to a eutrophic environment (i.e., harbors) has been assessed, and sights of early adaptation mechanisms to oligotrophic coastal waters have been explored. In the forthcoming scenario of a warmer Mediterranean Sea, to anticipate mitigation countermeasures against bio-invasions, it is of paramount importance to predict invasiveness potential of non-indigenous species by their ecological profiling through assessment of the key biological traits that may grant individual success in terms of growth and reproduction in a given ecosystem. Life in fluctuating environments requires species to evolve traits and feedforward strategies [67] ensuring ecophysiological plasticity, i.e., the ability to detect, predict, and adjust individual functioning, to ultimately cope with a changing environment. So far, information on the regulatory functioning of the photosynthetic machinery of the *Cassiopea* holobiome in response to light penetration constraints is missing. Here, changes in light penetration were reproduced in laboratory experiments to assess the physiological plasticity of the photosynthetic machinery of *Cassiopea* jellyfish in response to a simulated transition from a harbor-typical high turbidity condition to a meso-oligotrophic, coastal open water condition. In addition to its eurythermal tolerance [68, 69], our results indicate that *Cassiopea* autotrophic system is highly adaptive. Harbors and other anthropogenic sheltered areas can therefore act as initial settling terminals for upside-down early founder jellyfishes; also, it is foreseeable that the progressive northward displacement of the 15° isotherm will be paralleled by *Cassiopea* population spillover in the surrounding natural habitats of the Western Mediterranean Sea, as it already happened into the Levantine basin [47, 49].

Symbionts in *Cassiopea* specimens were identified as belonging to the *Cladocopium* genus (formerly clade C), the taxon with the highest species richness and the most ecologically and physiologically diversified within the family Symbiodiniaceae, hosted also by sponges, corals and other cnidarians, flatworms and bivalves, as well as by protists (ciliates and foraminiferans) [15]. In *Cassiopea*, *Cladocopium* is often associated to *Symbiodinium* and *Breviolum* (reviewed by Djeghri et al. [23]). Incubation at the two different light conditions (irradiance 200 vs 500 μmol photons m^-1^ s^-1^) did not produce a significant difference in symbiont abundance (Fig 1). Our results are in line with the observations by Mortillaro et al. [25] who did not detect significant change in symbiont density in jellyfish displaced from high (750 μmol photons m^-1^ s^-1^) to low light (~200 and ~75 μmol photons m^-2^ s^-1^) conditions, even after an incubation period > 2 weeks. This suggest that symbiont cell division is a process too slow to produce significant changes in symbiont abundances after only one-two week of exposure. However, our *Cassiopea* specimens exposed to enhanced irradiance (ELP) showed a lower content in chlorophyll *a* and *c*, and thus a lower Ci than in specimens exposed to reduced light conditions (RLP). The chlorophyll pool increment in RLP compared to ELP condition did not correspond to an enhanced photosynthetic capacity. These results demonstrate a highly efficient acclimation potential and photosynthetic plasticity of *Cladocopium* in response to changes in light conditions. Also, modifications in pigment concentration and composition (including production of photoprotecting pigments) represent a consequence of an exposure from hours to days to an enhanced light condition [70, 71] Our results therefore suggest that the cell size and chlorophyll content of symbiotic *Cladocopium* undergo rapid changes with short-time environmental variations. This ecophysiological plasticity represents a strong competitive advantage in habitats with rapid variations in the environmental conditions, such as harbors and sheltered waters, which may turn from turbid to transparent to turbid again within few hours. Indeed, the shift from high to low irradiances causes an increased chlorophyll content to harvest a higher quantity of light [25]; at the opposite condition, in response to enhanced light conditions, the photosynthetic pigment production is reduced to avoid photoinhibition [72]. Chlorophyll contents per symbiont (Ci) found in *Cassiopea* jellyfish are comparable to that observed in other Mediterranean cnidarians, such as the scleractinian *Cladocora caespitosa* and the octocoral gorgonian *Eunicella singularis* [73–75]. Also, the organic matter content of *Cassiopea*, mainly represented by proteins, ranged between 27% and 33% of the jellyfish dry weight, in line with values observed in other symbiotic jellyfish [76–79].

The different RLP or ELP irradiance conditions (200 vs 500 μmol photons m^-1^ s^-1^) did not influence the photosynthetic rates of *Cassiopea* specimens (Fig 3). The analysis of photosynthetic performances showed similar trends and photosynthetic parameters (Pmax, α, E_k_) between the two experimental groups. The saturation and the compensation irradiance levels were higher than previously recorded by Welsh et al. [56] on Australian *Cassiopea* sp.; in our experiment, in both RLP- and ELP- photosynthesis curves, saturation was reached at irradiance of 800 μmol photons m^-1^ s^-1^ (instead of 400 μmol photons m^-1^ s^-1^; [56]) and compensation was reached at 196 and 238 μmol photons m^-1^ s^-1^ under PAR 200 and PAR 500, respectively (instead of approx 50 μmol photons m^-1^ s^-1^; [56]). These variations might be due to the shorter dark incubation time in our experiment (20 minutes) compared to a longer incubation (overnight) in Welsh and colleagues [56]. With a longer dark incubation, as occurring at night within a circadian cycle, the light harvesting capacity can be reduced due to periodical oscillations of light-harvesting protein synthesis on the thylakoid membranes [80]. Photosynthesis to respiration ratio (P/R) can be used as proxy for autotrophic contribution [81]. Our findings (PAR = 500, P/R = 1.4; PAR = 200, P/R = 1.9) are in accordance with the values observed in *C*. *andromeda* (P/R = 1.4) [82] and in other zooxanthellate jellyfish such as *Linuche* and *Mastigias* (P/R of 1.7 and 1.1–1.8 respectively) [31, 32]. Nevertheless, much higher P/R values were found in *Cassiopea* sp. from Australian shallow waters (P/R of 2.04; [56]). In all cases, P/R>1 indicates that the production of symbiotic photosynthates overcome the oxygen consumption so that the autotrophic input may be used for sustaining both metabolic maintenance costs and growth of the host, as the symbiont organic carbon production can satisfy up to 169% of the host respiratory demand (CZAR) [20]. Rapid light-response curves (RLCs) represent the short-term (few minutes) actual photosynthetic capacity of photosystem II [61]. The high photosynthesis rates of *Cassiopea sp*. are confirmed also by its photosynthetic efficiency measured as relative electron transport rate (ETR), a relationship not always observed in corals [83]. As showed in Fig 4A, RLCs showed no saturation of the ETR at maximum light natural condition (2000 μmol photons m^-1^ s^-1^), in line with the observation by Jantzen et al. [29], indicating there is no photoinhibition of photosystem II [84]. The unsaturation of the ETR in our experiment is not fully reflected by the P-I curve, which indeed shows a saturation point, reaching a plateau. The mismatch between respirometry and fluorometry has been studied mostly for corals [75]; however, differences in the photosynthetic performances of symbiotic anthozoans may be due to the ecological plasticity of the symbiont clades shared among cnidarians, in tropical as well as temperate environment [85, 86]. In our experiment, the respirometry- fluorometry relationship was not linear, except at moderate light. This result indicates that less oxygen was produced per charge separation under high light. The nonlinearity could be due to an under-estimation of the light-enhanced respiration rates of the animal [87] or to non-photosynthetic electron transport [88]. Such process can be due to the development of the Mehler cycle or photorespiration processes or to a cyclic flow of electrons around PSII [89]. Cyclic electron transport could play an important role in photoprotection as it has been observed for diatoms [90, 91]. Rapid light curves (RLCs), showed an increase with increasing light intensities (both experimental conditions) in the non-photochemical quenching (NPQ) which measures the thermal dissipation of the excess excitation energy. Conversely, the quantum yield (Y) showed a decrease with increasing irradiances, underlying that the incoming energy is channeled into the non-photochemical pathway. The same behavior was observed in other Cnidaria from the Mediterranean Sea, such as *Cladocora caespitosa* [75]; however, further studies are essential to understand *Cassiopea* symbionts light tolerance through relaxation kinetics.

Thus, no matter the acclimation period at low light level, in our experiment, *Cassiopea* jellyfish maintained high photosynthetic efficiency (confirmed by RLCs curves). This may be interpreted as the potential of the upside-jellyfish to easily withstand different light conditions, and to colonize a wide range of shallow water environments. Therefore, it is likely that in habitats with reduced light penetration conditions (e.g., harbors, marinas) a basal photosynthesis is still carried out even if the main trophic strategy is based on heterotrophic resources. As a corollary hypothesis, it is possible to speculate that summer surface heating of confined habitats, such as harbors, may support introduction and establishment of warm-water affinity species, such as *Cassiopea*. Within the current scenario of a warming Mediterranean Sea, the upside-down jellyfish seem to possess suitable requisites to spill over from harbors into open coastal waters, where high symbiont photosynthesis will be maintained also at increased irradiance levels.

Shifts between autotrophy and heterotrophy have been studied in temperate scleractians corals and gorgonians. While corals seem to shift their nutrition mode depending on the season, gorgonians are able to maintain in parallel both strategies [73, 86]. In *Cassiopea* sp., only the shift from autotrophy to heterotrophy has been studied so far [25], showing how photosynthesis in an essential part of the autoecology of the species even at low light level (75 μmol photons m^-1^ s^-1^). Under that condition, no shift towards heterotrophy was observed (through fatty acid transfer analysis) and shrinking of the medusa occurred. However, in order to better understand the relative contribution of autotrophy and to what extent jellyfish can rely on heterotrophy, carbon translocation should be quantified by stable isotopes analyses (e.g., [92]). Further translocation studies could shed light in the autotrophic-heterotrophic balance of the species.

In conclusion, the photosynthetic performance of *Cassiopea* was high—no matter the acclimation–even in environments where photosynthesis is not well supported, such as turbid harbor habitats. Harbors as well as other artificial, coastal habitats, as coastal shrimp farms, may represent suitable environments for *Cassiopea* settlement and rapid growth by sustaining a constant, heterotrophic jellyfish metabolism [51]. In the present work, we tested the ability of *Cassiopea* sp.to react to a change towards meso-oligotrophic waters. Variable light exposure is only one of the environmental factors that plays a role in the population establishment and persistence. However, the ability to cope with fast changes in abiotic conditions, such as light exposure and temperature, together with their dual nutrition mode make the upside-down *Cassiopea* potential winners in a changing Mediterranean Sea scenario, to the detriment of less tolerant taxa, such as indigenous species, which are already struggling with these sudden changes.

## Supporting information

S1 Fig Chlorophyll and symbionts. (A) Chlorophyll a (B) Chl c_2_ and (C) Total chl and (D) Symbionts count and (E) Chlorophyll content per symbiont of *Cassiopea* based on two different light conditions (RLP and ELP). Data are normalized to DW. Data represent mean *±* SD, n = 20.
(TIF)

S2 Fig *Cladocopium*-*Cassiopea* photobiological performance (net photosynthesis vs. irradiance). Data are normalized to AFDW. Each point represents means *±* SD, n = 20. PAR 200R^2^ = 0.976; PAR 500 R^2^ = 0.976.
(TIF)

S3 Fig Rapid light curves (RLCs). (A) Relative electron transport rate (rETR) (B) Non-photochemical Quenching (NPQ) (C) Yield of PSII (Y) of *Cassiopea* at the different light conditions (RLP and ELP) measured from the sub-umbrella. Data represent mean *±* SD, n *=* 20.
(TIF)
