# Supplementary figures and images for: High photosynthetic plasticity may reinforce invasiveness of upside-down zooxanthellate jellyfish in Mediterranean coastal waters
Source: PLoS One. 2021 Mar 19;16(3):e0248814. doi: 10.1371/journal.pone.0248814 (PMC7978352; doi:10.1371/journal.pone.0248814)

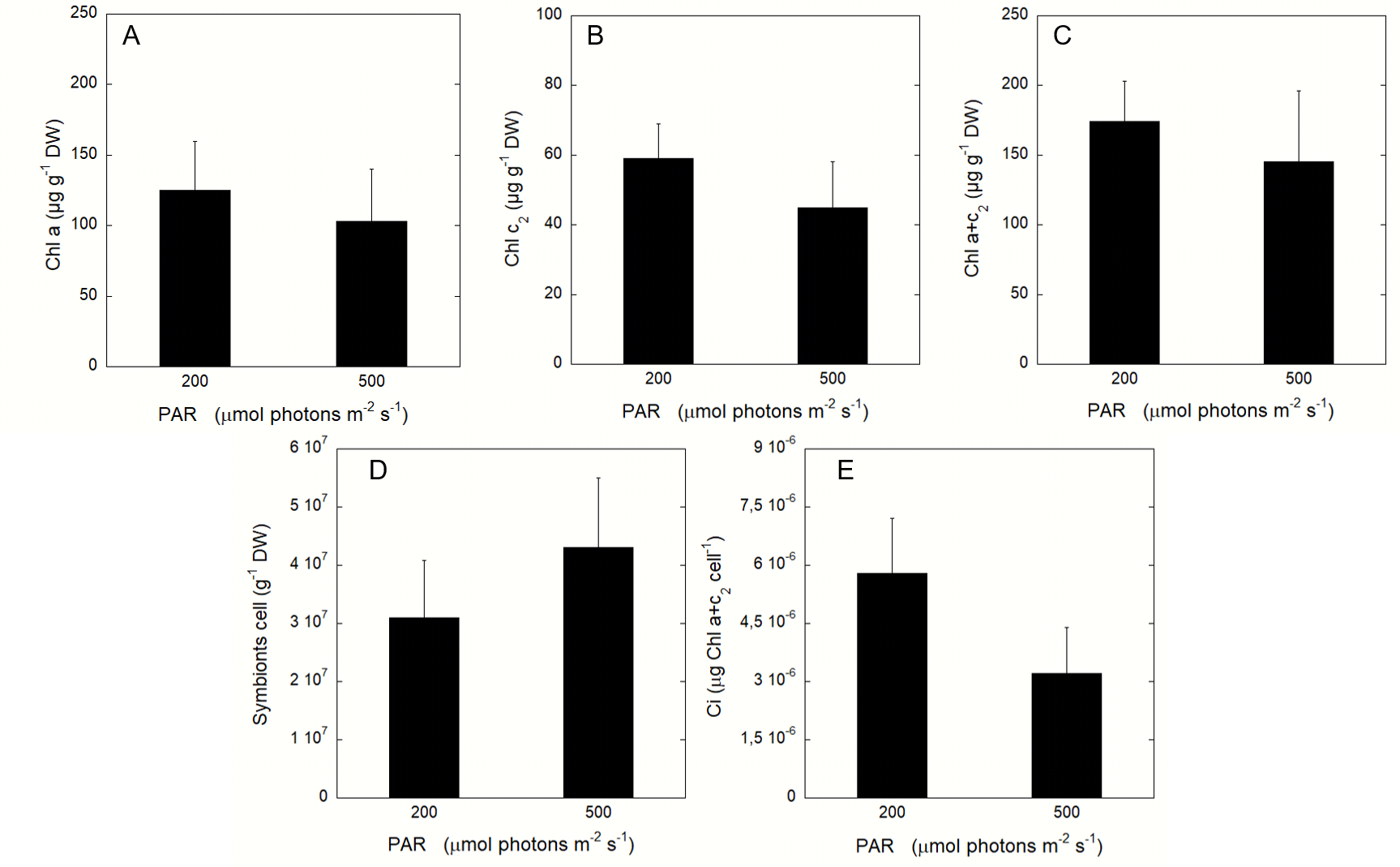

Supplement: S1 Fig — (A) Chlorophyll a (B) Chl c2 and (C) Total chl and (D) Symbionts count and (E) Chlorophyll content per symbiont of Cassiopea based on two different light conditions (RLP and ELP). Data are normalized to DW. Data represent mean ± SD, n = 20. (TIF) [file pone.0248814.s001.tif]

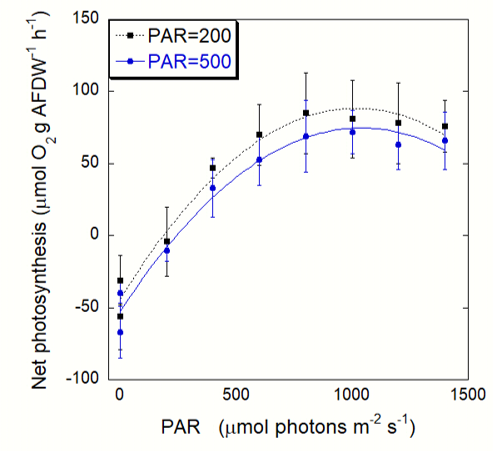

Supplement: S2 Fig — Data are normalized to AFDW. Each point represents means ± SD, n = 20. PAR 200R2 = 0.976; PAR 500 R2 = 0.976. (TIF) [file pone.0248814.s002.tif]

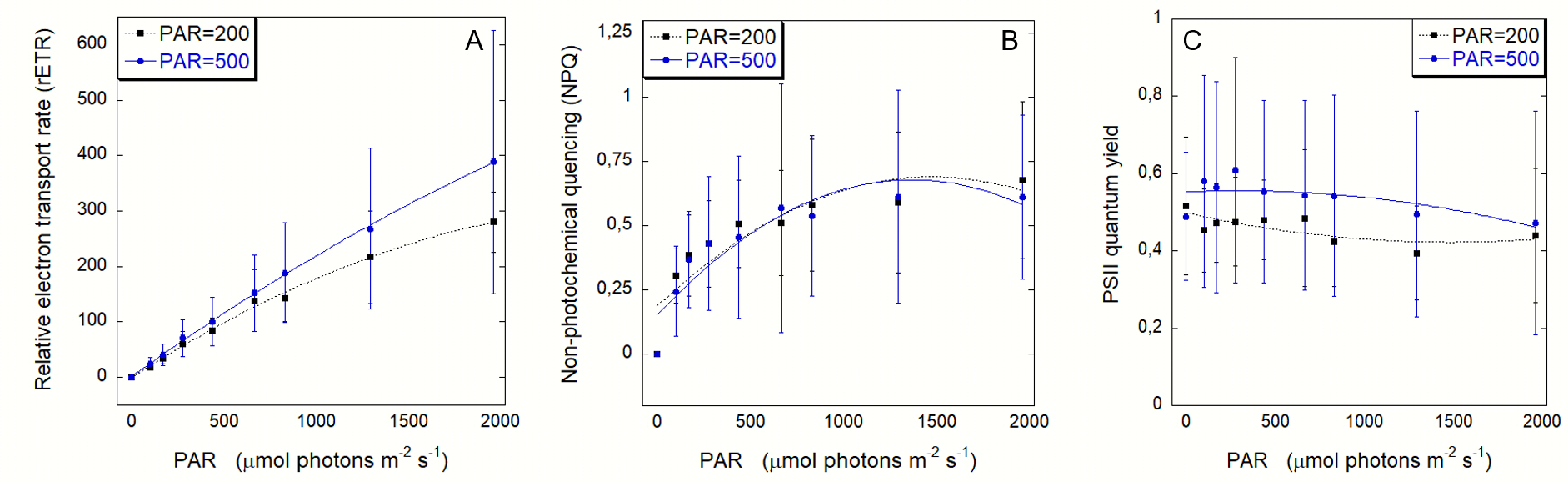

Supplement: S3 Fig — (A) Relative electron transport rate (rETR) (B) Non-photochemical Quenching (NPQ) (C) Yield of PSII (Y) of Cassiopea at the different light conditions (RLP and ELP) measured from the sub-umbrella. Data represent mean ± SD, n = 20. (TIF) [file pone.0248814.s003.tif]
